# The retention of training doctors in the Irish health system

**DOI:** 10.1007/s11845-023-03288-8

**Published:** 2023-02-16

**Authors:** Tom Pierse, Roisin Morris, Leah OToole, Brian Kinirons, Eddie Staddon

**Affiliations:** National Doctors Training and Planning (NDTP), Health Service Executive (HSE), Dublin, Ireland

**Keywords:** Doctor migration, Retention, Workforce planning

## Abstract

**Background:**

There is limited quantitative evidence on the migration patterns of training doctors in Ireland. The aim of this study is to estimate the number of trainee doctors leaving the Irish health system and the numbers returning.

**Methods:**

This study uses administrative data to track the migration patterns of Irish trained doctors at various career stages.

**Results:**

Eighty-four percent of interns who commenced intern training in 2015 subsequently commenced a basic specialist training (BST) or general practice (GP) training programme in subsequent years (2016–2021). Of those who completed BST training in 2017, 75% went on to higher specialist training (HST) in Ireland. In 2021, of the 2016 cohort of doctors awarded Certificates of Satisfactory Completion of Specialist Training (CSCST), 68% are employed in Ireland and 32% are abroad or unknown. Of the 2016 group that are abroad, the UK is the main country of practice. There are variations in the retention rate across disciplines; from the 2016 cohort, 52% of anaesthesiology CSCSTs were working in Ireland in 2021 compared to 88% of psychiatry CSCSTs.

**Conclusion:**

Previous research has highlighted Irish doctor’s intentions to migrate and intentions to return to Ireland. This study documents for the first time the extent to which Irish doctors are leaving and returning to the Irish health system from 2015 to 2021. The paper also gives a picture of variations across medical disciplines and the location of emigration of qualified specialists.

## Introduction

Concerns about the outward migration of doctors in high-income countries have been noted in a range of countries [1–4]. Relative to other European countries, Ireland, the focus of this study, has low rates of consultants per capita [5] and is heavily reliant on doctors from other countries for service delivery, particularly in some regional (model 3) hospitals [6]. Under the WHO Global Code of Practice on the International Recruitment of Health Personnel, countries are obliged to be self-sufficient in its production of healthcare workers. Ireland also has the highest number of medical graduates per capita in the OECD [7]. In order to have suitably trained applicants for new and replacement consultant posts, a sufficient number of specialists need to be trained and retained. In this study, we analyse the number of training scheme doctors leaving the Irish health system and the numbers that return. The number of training scheme doctors that are temporarily and permanently leaving the Irish health system will have an important influence on the number of trainees available to staff hospitals and the number of applicants for consultant posts.

International research indicates that, at an aggregate level, Ireland has a relatively high emigration rate compared to other countries [8]. Previous work in Ireland has used the professional registers and/or immigration and census records from a small number of countries to estimate the scale of migration among Irish doctors [9, 10]. Earlier research by the HSE had indicated that more than half of the doctors who completed their internship in Ireland in 2011 had migrated within the year [11].

Doctors’ migration intentions have also been used to indicate the potential scale of migration. One approach is to use the number of verification certificates issued by the Medical Council of Ireland as an indicator of the number of doctors leaving the Irish health system [9]. To migrate from Ireland, doctors must have their registration on the Irish register of medical practitioners verified by the Medical Council of Ireland prior to migration. While this data does not show that a person migrated, it does show some level of intent. This data shows that there were 1881 verification certificates issued by the Medical Council in 2015 [9]. More recently, a large survey of junior doctors showed that 45% of respondents planned to remain in Ireland, 35% planned to leave but return later, 17% planned to leave and not return and 3% planned to quit medicine [1]. A survey of trainee doctors found that, of those intending to migrate, Canada, the UK and Australia were the most popular destinations [12]. A survey of Irish medical students found that 88% would potentially migrate following the intern year [13].

The intention to go abroad is strongly associated with nationality—non-EU doctors are most likely to have an intention to leave and not return [1]. Ireland has the highest number of medical graduates per population among OECD countries; however, approximately 50% of medical students are international, mainly fee-paying, students [7]. The intention to go abroad is also associated with a negative experience of mentoring which suggests that the quality of training and the way in which it is provided are important determinants of doctor’s intention to remain in the Irish healthcare system [1].

Qualitative research of trainee doctors and doctors who have migrated has indicated a number of reasons for people leaving Ireland, including career progression and working conditions [9, 14]. Most specialties, with the exception of general practice (GP), encourage trainees to migrate to achieve career progression (ibid).

This study uses a novel panel dataset of training and consultant doctors to track the career progression of Irish training doctors over time. This allows us to document when doctors leave the Irish public health system and if they return in subsequent years. This analysis is supplemented with a web search to identify the current place of work of the doctors that are currently not working in the Irish public health system; this allows us to identify qualified specialists who trained in Ireland who are currently working in the private sector in Ireland or abroad.

## Methods

The number of consultants, training and non-training scheme hospital doctors is sourced from Doctors Integrated Management E-System (DIME), a comprehensive administrative database maintained by the national health provider, the Health Service Executive (HSE). DIME is a quadripartite system which encompasses National Doctors Training and Planning (NDTP), the Medical Council of Ireland, the Postgraduate Medical Training Bodies and Clinical Sites. DIME records registration, training and employment details of all consultant and non-consultant hospital doctors (NCHDs) in Ireland who are employed in the public service. DIME includes doctor’s medical council numbers which can be used as a unique identifier over time; when doctors leave and return after several years, the same medical council number is maintained.

**Fig. 1:**
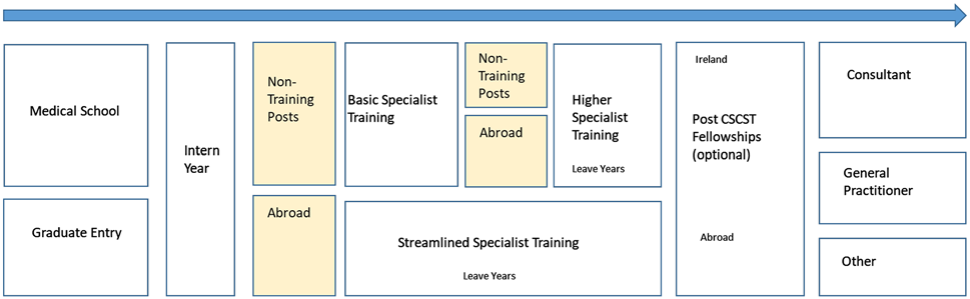
Career pathways of doctors in Ireland

Figure 1 outlines the typical career pathways of doctors in Ireland. The duration of training for doctors is long; following a year of intern training, doctors typically complete 2 years of basic specialist training (BST) and 2 to 6 years of higher specialist training (HST) after which they are awarded a Certificate of Satisfactory Completion of Specialist Training (CSCST). Following CSCST, doctors (with the exception of GPs) frequently do 1 to 2 years of fellowship training. In addition, doctors may go abroad, work in non-training scheme posts in Ireland between training stages or take leave years. For this reason, given that the data is available from 2015, a staged approach was used to show the retention rates between the training stages. Retention rates are shown between the intern year and BST training, BST training and HST training, and finally between CSCST and consultant posts.

Data was accessed from 2015 (the DIME databased commenced in 2015) to 2021, for October of each year. Trainees are allocated to cohorts based on the year of commencement, and year of completion, of their programme. The DIME records include information on trainees who were in the middle of a training programme when the data collection process started in 2015; thus, the analysis includes cohorts of higher specialist trainees (HSTs) with start dates from 2013. GP trainees are separately identified in the records due to the different duration of training. DIME data is supplemented with lists of trainees who had been awarded CSCST and BST completion, sourced from the postgraduate training bodies.

The main analysis in this study uses DIME data from 2015 to 2021. However, a targeted web search was carried out of CSCSTs who graduated in 2016 and 2017 and who were no longer recorded in the DIME system. These earlier years were used as doctors were more difficult to find using this method in later cohorts. The aim of this search was to establish the country of current practice of this cohort. The main sources of information included LinkedIn, hospital websites, NHS websites and affiliations on recent academic publications. While this method is not definitive, it gives a clear indication of the current location of practice of these doctors.

## Results

### Interns

Table 1 shows the progression of interns in the 3 years after internship. The table shows the number of interns in each year and the proportion that are still in the Irish public health system in subsequent years. The table shows that in the year following the intern year, on average, 32% of trainees are on a BST programme, 21% are in non-training roles and 47% have no record. The latter indicates that almost half of interns do not practise in the Irish public health system in the year following internship. In the second year after internship, the proportion of interns in BST programmes increases from 32 to 49% on average. By year 3, the proportion of interns having started a BST programme is 69% on average.

**Table 1:** Number of interns in the Irish public health system in subsequent years

**Year**	**Interns**	**y + 1**						**y + 2**							**y + 3**	
		Started BST or GP training in y + 1		Non-training y + 1		No record y + 1		In BST or GP training in y + 2		Non-training y + 2		No record y + 2			Started BST or GP in *y* + 1, y + 2 or y + 3	
**2015**	668	260	39%	94	14%	314	47%	382	57%	35	5%	251	38%	483		72%
**2016**	693	236	34%	108	16%	349	50%	360	52%	37	5%	296	43%	487		70%
**2017**	692	215	31%	133	19%	344	50%	311	45%	41	6%	340	49%	460		66%
**2018**	717	215	30%	128	18%	374	52%	359	50%	56	8%	302	42%	467		65%
**2019**	731	235	32%	249	34%	247	34%	311	43%	65	9%	355	49%			
**2020**	966	256	27%	239	25%	471	49%									
**Mean**			**32%**		**21%**		**47%**		**49%**		**7%**		**44%**			**69%**

In the second year after internship, the number of doctors in non-training scheme posts reduces from 21 to 7% and the proportion who are not working in the Irish public health system fall from 47 to 44%.

Table 2 shows the progression of interns in each cohort year to 2021. The table shows that for the 2015 and 2016 intern cohorts, 84 and 81%, respectively, started BST or GP training prior to 2021. As is to be expected, this proportion is higher in earlier cohorts. This is due to interns temporarily leaving the public health system for one or more years before returning to commence a further training programme.

**Table 2:** Training progression and non-training years of intern cohorts by 2021

**Year**	**Interns**	**Started BST or GP training**	**%**	**Non-training record only**	**%**	**No subsequent record**	**%**
**2015**	668	558	84%	23	3%	87	13%
**2016**	693	563	81%	33	5%	97	14%
**2017**	692	520	75%	39	6%	133	19%
**2018**	717	467	65%	52	7%	198	28%
**2019**	731	328	45%	206	28%	197	27%
**2020**	966	256	27%	239	25%	471	49%

There are a proportion of interns that go on to non-training posts and do not progress to further training, comprising 3–5% of the 2015–2016 cohorts. In addition, there are 13–14% of interns who left the Irish health system after internship and did not return.

Together, Table 1 and Table 2 show that while many interns leave the Irish public health system after internship, the vast majority subsequently return.

### Basic specialist training

Table 3 shows the extent to which trainees who complete BST training (excluding GPs as recorded separately) go on to further training in the 2 years following completion of a BST training programme. In the training year following completion of BST training, on average between 2017 and 2020, 55% of trainees go directly into a HST or GP training programme. A further 21% are on non-training scheme years, and there is no record of 23% of trainees. The latter suggests they may have left the country, are working in the private sector, in research, or are not working in a clinical role. In the second year after completing BST, the proportion in HST rises to 65% with the number in non-training roles falling to 11%.

**Table 3:** BST completions in subsequent years

**Year**	**Complete BST**	**y + 1**						**y + 2**					
		In HST or GP training*		Non-training		No record		In HST or GP training*		Non-training		No record	
**2017**	381	201	53%	91	24%	83	22%	234	61%	46	12%	96	25%
**2018**	445	234	53%	91	20%	111	25%	289	65%	55	12%	95	21%
**2019**	427	254	59%	82	19%	88	21%	295	69%	41	10%	88	21%
**2020**	450	248	55%	91	20%	105	23%						
**Average**			55%		21%		23%		65%		11%		22%

*A small number of BST completions go on to start a different BST programme

Table 4 shows the extent to which trainees who complete BST training go on to further training, in either general practice or HST by 2021. Of the 2017–2019 cohorts, approximately three-quarters of trainees had progress to further training by 2021. Data on the completion of BSTs, primarily sourced from the postgraduate training bodies, is not fully recorded for 2015 and 2016 and thus is not shown.

**Table 4:** BST completions that start HST or GP training

**Year**	Completed BST*	**Start HST or GP training by 2021**	**%**
**2017**	381	286	75%
**2018**	445	319	72%
**2019**	427	308	72%
**2020**	450	248	55%

*Excludes anaesthesiology (streamlined) and GP trainees (recorded separately). Trainees who completed BST started in range of years due to differences in the duration of programmes and leave

There are fewer HST places than BST trainees each year resulting in competitive pressures, which vary across the medical disciplines. Table 5 shows the variation across the disciplines in the number and proportion of doctors who having completed BST in 2017 going on to further training by 2021. These range from 68 (medicine) to 88% (emergency medicine).

**Table 5:** Progression of complete BSTs by medical discipline 2017*

**Medical discipline**	**Completed BST**	**Progressed to HST or GP by 2021**	**%**
**Emergency medicine**	24	21	88%
**Medicine**	203	139	68%
**Obstetrics and gynaecology**	20	15	75%
**Ophthalmology (medical and surgical)**	10	5	50%
**Paediatrics**	38	33	87%
**Pathology**	20	16	80%
**Psychiatry**	19	16	84%
**Surgery**	47	41	87%
**Total**	381	286	75%

*Anaesthesiology was excluded as it is fully streamlined. Surgery was included as it is not purely streamlined

The discipline of medicine is of particular interest as it supplies trainees for a number of HST training programmes. Table 6 shows the number and proportion of doctors who complete BST in medicine who then go on to further training. The table shows that 53% of BSTs in medicine go on to do a HST in the discipline of medicine. Other major pathways for BSTs in medicine are general practice, radiology and pathology. However, BST in medicine does not qualify you to progress to HST in certain specialties such as obstetrics, psychiatry, ophthalmology and surgery; they have to go back to do BST.

**Table 6:** Progression of BST Medicine 2017 cohort in 2021

**Medical discipline**	**No**	**%**
**Medicine**	73	53%
**General practice**	36	26%
**Radiology**	15	11%
**Pathology**	12	9%
**Other**	3	2%
**Total**	139	100%

### Qualified specialists

#### Consultants

Table 7 shows the number of CSCSTs by year of award (excluding GPs, public health and occupational health specialists) and their status in 2021. Overall, 61% of the 2016 CSCST cohort was employed in a public consultant post in 2021. The proportion of each cohort in a consultant post declines for the more recent cohorts as is to be expected; for the 2021 cohort, 14% were in a consultant post. A small number of recently qualified specialists work as NCHDs in the public health system.

**Table 7:** Number of CSCST completions by year

**Year**	CSCST*	**Public consultant post** **in 2021**	**%**	**NCHD in 2021**	%	**Not in Irish public health system**	**%**
**2016**	212	130	61%	0	0%	82	39%
**2017**	182	109	60%	2	1%	71	40%
**2018**	229	113	49%	3	1%	113	49%
**2019**	185	81	44%	2	1%	102	55%
**2020**	151	33	22%	8	5%	108	73%
**2021**	209	29	14%	47	22%	131	64%

*Excludes GPs public health and occupational health

#### Consultant CSCSTs—current location

Table 8 shows the CSCST cohorts from 2016 to 2017. Of the 212 CSCSTs in the 2016 cohort, 130 were employed in a consultant post in the Irish public health system by 2021. Of the 82 doctors from this group not in the Irish public health system in 2021, a web search indicated that 15 were likely to be practising in Ireland in the private sector and 67 were abroad or location unknown. This pattern is repeated for the 2017 cohort. Including public and private consultants, there are an estimated 68% of the 2016 cohort and 64% of the 2017 cohort working as consultants in the Irish health system in 2021.

**Table 8:** CSCST 2016 and 2017 Cohorts—current location

**Year**	**CSCST**	**Public consultant 2021 (DIME)**	Total Ireland*	**Abroad/unknown**
**2016**	212	130	145	67
		*61%*	*68%*	*32%*
**2017**	182	109	117	65
		*60%*	*64%*	*36%*

*Includes private sector

**Fig. 2:**
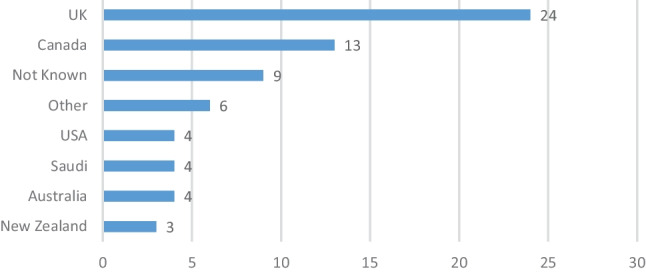
Current country of practice of 2016 cohort not in Ireland

Figure 2 shows the probable current country of practice of the 2016 CSCST cohort that are not in Ireland. The figure shows that the UK is the most frequent country of current practice for this group.

#### CSCST by medical discipline

Table 9 shows the retention rates by medical discipline for the 2016 and 2017 cohorts. The table shows a wide variation in the retention rates across the disciplines. While there is a degree of variation across the 2 years, disciplines such as anaesthesiology and medicine have lower retention rates compared to psychiatry and surgery.

**Table 9:** Number of CSCSTs (2016 cohort) in public consultant posts by medical discipline

**Medical discipline**	**CSCST 2016**	**Consultant post (public or private)** **2021**	**%**	**CSCST 2017**	**Consultant post (public or private)** **2021**	**%**
**Anaesthesiology**	56	29	52%	36	22	61%
**Emergency medicine**	4	4	100%	0	0	NA
**Medicine**	62	41	66%	57	32	56%
**Obstetrics and gynaecology**	8	8	100%	5	4	80%
**Paediatrics**	12	10	83%	14	7	50%
**Pathology**	15	11	73%	13	11	85%
**Psychiatry**	16	14	88%	19	18	95%
**Radiology**	19	11	58%	15	4	27%
**Surgery**	20	17	85%	23	19	83%
**Total**	212	145		182	117	

#### GP CSCSTs

Table 10 shows the proportion of GP trainees that have received CSCST by 2021. Of the cohort starting GP training in 2015, 94% had completed by 2021. Of the cohort starting GP training in 2016, 80% had completed by 2021. Given that GP training is a 4-year-training course, this indicated a substantial portion of trainees take longer than 4 years to complete.

**Table 10:** Number of GP CSCST completions by year

**Year**	**Start GP** t**raining**	**CSCST by 2021**	**%**
**2015**	195	184	94%
**2016**	175	140	80%
**2017**	170	105	62%

## Discussion

Previous research in Ireland has focused on the migration intentions of trainee doctors [1]. In this study, we use an administrative panel dataset to show the movement of doctors through the training stages, non-training scheme years, outward migration and return. Our findings confirm what is widely believed but which had not previously been fully documented that there are high levels of outward migration of Irish doctors at different stages along their career path. Our findings also show that the majority of doctors return following a period out of the Irish public health system. This is an important finding that has previously been underemphasised.

Consistent with previous research [11], the analysis shows (Table 1) that a large proportion of interns do not enter basic specialist training directly after internship. In the year following the intern year, 47% of interns temporarily leave the Irish public health system; a substantial numbers of Irish doctors have been shown to travel to Australia [10]. However, what the data presented in this paper demonstrates is that following a period out of the Irish public health system, most interns (84% of the 2015 cohort) do return to take up a training position (Table 2)

Our findings show that following completion of a BST programme, approximately three-quarters of BSTs go on to either HST or GP training (Table 4). However, there are substantial variations in the rates of retention across the medical disciplines (Table 5). For surgery, paediatrics and emergency medicine, approximately 87% of BST completions go on to HST training in Ireland. However, medicine, a large discipline, has a retention rate of 68%. A mismatch between the number of BST and HST places is likely to be a significant factor resulting in a lower retention of BST medicine trainees. BST medicine trainees go on to a range of specialty training programmes (Table 6); 53% of those who go on to further training in Ireland do HST in medicine, while a further 26% go on to GP training in subsequent years. In addition, some specialties, for example, within pathology and radiology, do not have a dedicated BST training programme, and BST in general medicine is one route into these specialties.

Our findings show that non-training years are common after BST training (Table 3). For example, in the year after completing BST training, 21% of doctors go on to one or more years in non-training posts (including research posts), in the Irish public health system; the number of doctors in non-training scheme years after BST may represent the group who were unsuccessful at their first attempt at competitive HST application. Current policy in Ireland is to identify opportunities to shortening the length of time from internship to CSCST [15]. One approach to this is to introduce streamlined programmes to shorten the total training duration, thus eliminating “gap years” between basic and higher training, and also reduce attrition. While some specialties are fully streamlined, such as general practice and anaesthesiology, others are transitioning. One of the requirements to implement stream lining is sufficient places at HST to allow flow through from BST to HST. Streamlined training may not be suitable for some disciplines due to the variety of training pathways. In these cases, a closer alignment between BST and HST training intakes through an increase in the HST intake may reduce the need for gap years.

For doctors who complete CSCST, the majority leave the public health system (Table 7) for fellowship opportunities. For example, of the 2020 cohort of CSCSTs, 22% were working in the Irish public health system in 2021.

However, most do return. The best picture of this can be seen from the 2016 cohort; by 2021, 61% were working in the public health system in Ireland. In addition, a further 7% of the 2016 cohort are working in Ireland in the private sector. However, substantial variations across the medical disciplines are shown (Table 9). The retention of fully trained specialists is of particular concern. These doctors represent a large investment by the state. There are significant benefits to having some doctors receive further training and experience abroad such as access to higher volumes of practice in their subspecialty area, latest techniques and the opportunity to undertake research. However, there are roles with lower levels of specialisation, for which foreign training may not be as necessary. For these roles, the risk of doctors becoming settled abroad may outweigh the benefit of a higher level of specialist experience.

The level of retention of CSCST doctors is a key factor in the determination of the number of HST training posts to be provided. However, the level of retention of Irish-trained CSCSTs documented here needs to be viewed in the context of available consultant posts for each specialty and special interest at the time when candidates are seeking posts in Ireland. While there is currently a substantial number of vacant consultant posts, most of these reflect posts that have been recently created [6]. Further research is required to understand the different drivers of retention rates, including the availability of posts, for each of the medical disciplines and specific specialties.

There are few studies comparing international comparing levels of retention internationally. One such study showed that Ireland has a comparatively high rate of emigration among both Irish-trained and Irish-born doctors [8]. There are a range of non-pay-related approaches which could be used to improve the retention of doctors in the Irish health system [16, 17]. These include increasing the number and attractiveness of new consultant posts; proleptic appointments; policies to support recruitment in smaller and medium-sized hospitals [18] or underdeveloped services; policies to support generalist training [19]; post-CSCST fellowships and increasing medical school places for Irish graduates of Irish schools.

There are a number of limitation to this study. The number of consultants in private hospitals is not centrally recorded. The web search method used in this study to identify consultants working in the private sector is not definitive and highlights the need for better data sources for the private sector. The quality of data held on DIME have improved over time with increasing site compliance and data validation exercises; however, missing records are likely in the earlier years of the database. The level of competition experienced by applicants for higher specialist training is a function of their citizenship status due to prioritisation rules; an analysis of competition ratios, the ratio of BST completions to higher specialist training places, is beyond the scope of this study. The data analysed in this study is from 2015 to 2021; due to the long training period for doctors, we show the extent to which doctors at various stages progress to the next stage, in the short term, until longer time series data is available; further simulation analysis will be required to estimate the overall retention rate across the full training programme.

Medical workforce planning requires a balance between neither training too many nor too few doctors, relative to the required number of new and replacement consultant posts required in the future. Oversupply will result in a waste in investment as trained doctors go abroad and do not return due to a lack of available consultant positions in Ireland; conversely training too few doctors will result in an insufficient supply of adequately trained specialists for consultant posts. This paper provides important evidence for projecting future flows of doctors through the training systems and on to consultant posts. The intention is to continue monitoring the retention rates of doctors to update these projections. There are a range of policy responses that can be employed to improve the retention of Irish-trained doctors. While some of these policies may take many years to bear fruit, others can be expected to have a short-term impact.

## Data Availability

The data that support the findings of this study were provided by the Health Service Executive and are not currently publicly available. Data are, however, available from the authors upon reasonable request and with the permission of the Health Service Executive.
